# Effects of Laser Applications on Fibroblasts Cultured on Zirconia Surfaces—A Systematic Review

**DOI:** 10.3390/jcm14248668

**Published:** 2025-12-07

**Authors:** Jacek Matys, Natalia Struzik, Agnieszka Kotela, Zuzanna Majchrzak, Julia Kensy, Marzena Laszczyńska, Witold Świenc, Agata Małyszek, Zbigniew Rybak, Maciej Dobrzyński

**Affiliations:** 1 Dental Surgery Department, Wroclaw Medical University, Krakowska 26, 50-425 Wroclaw, Poland; witold.swienc@umw.edu.pl; 2 Department of Nutrition and Drug Research, Faculty of Health Sciences, Institute of Public Health, Jagiellonian University Medical College, Skawińska 8, 31-066 Krakow, Poland; natalia.struzik98@gmail.com; 3 Medical Center of Innovation, Wroclaw Medical University, Krakowska 26, 50-425 Wroclaw, Poland; kotela.agnieszka@gmail.com (A.K.); zuzanna.h.nawrocka@gmail.com (Z.M.);; 4 Department of Pediatric Dentistry and Preclinical Dentistry, Wroclaw Medical University, Krakowska 26, 50-425 Wroclaw, Poland; julia.kensy@student.umw.edu.pl; 5 Department of Biostructure and Animal Physiology, Wrocław University of Environmental and Life Sciences, Kożuchowska 1, 51-631 Wrocław, Poland; agata.malyszek@upwr.edu.pl; 6 Pre-Clinical Research Centre, Wroclaw Medical University, Bujwida 44, 50-345 Wroclaw, Poland; zbigniew.rybak@umw.edu.pl

**Keywords:** biocompatibility, fibroblasts, laser irradiation, soft-tissue integration, zirconia

## Abstract

**Background/Objectives:** This systematic review aimed to evaluate and summarize the available evidence on the effects of light-based applications, including laser irradiation on fibroblast responses to zirconia surfaces. **Methods:** A comprehensive electronic search was performed in PubMed, Scopus, Web of Science, Embase, and WorldCat databases. After duplicate removal and eligibility screening, 17 studies met the inclusion criteria. Only in vitro and animal studies assessing fibroblast behavior on zirconia after light- or laser-based surface irradiation were included. Due to heterogeneity in study designs and parameters, data were qualitatively synthesized. **Results:** All included studies confirmed the biocompatibility of laser-modified zirconia surfaces. Various laser systems—including Er:YAG, Er,Cr:YSGG, Nd:YAG, diode, excimer, and femtosecond lasers—were investigated. Most studies reported enhanced fibroblast adhesion, proliferation, and cytoskeletal organization compared with untreated controls. Two of the included studies demonstrated an antibacterial effect of erbium lasers treatment on zirconia surfaces. However, outcomes varied depending on the laser parameters, irradiation energy, and zirconia type used. **Conclusions:** Laser-based surface modification of zirconia appears safe and biocompatible, with evidence indicating favorable effects on fibroblast adhesion, proliferation, and organization. While these findings are promising for optimizing soft-tissue integration around zirconia implant abutments, further standardized and long-term studies are necessary to determine optimal laser settings and confirm clinical applicability.

## 1. Introduction

Implant dentistry is now regarded as a reliable approach for replacing missing teeth, providing predictable functional restoration and satisfactory esthetic outcomes. Titanium remains the most commonly used implant material because of its excellent success rate and long-term clinical performance [1,2,3,4]. However, concerns related to esthetics and soft-tissue reactions have stimulated research into alternative materials [5,6,7,8,9]. Zirconia dental implants have attracted increasing attention as a substitute for titanium, especially in regions of the mouth with high esthetic demands [2,3,10,11,12,13,14,15,16]. In addition to their superior esthetic properties, zirconia exhibits favorable biocompatibility and a lower affinity for bacterial adhesion compared with titanium, which can contribute to maintaining peri-implant health [10,11,17,18,19,20,21,22]. Long-term success depends not only on osseointegration but also on the stability of the peri-implant mucosal barrier [10,23,24]. Fibroblasts form the first cellular line of defense at the implant–mucosa interface by synthesizing and organizing collagen fibers, thus supporting connective tissue attachment and protecting peri-implant tissues [1,25,26,27,28].

**Figure 1:**
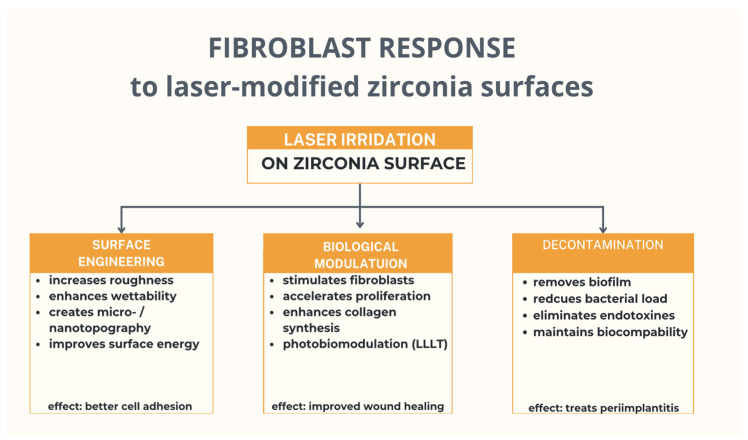
Applications of lasers in implant dentistry, including surface engineering, biological modulation, and decontamination.

However, successful implantation also depends on various other factors, one of the most critical being the implant’s surface topography [29,30,31,32,33], which influences primary stability, soft-tissue sealing, and marginal bone level maintenance [34,35,36]. Several techniques have been developed for modifying the Y-TZP (Yttria-stabilized Tetragonal Zirconia Polycrystal) surface, including (1) sandblasting (airborne particle abrasion), (2) acid etching (using hydrofluoric, nitric, or sulfuric acid), (3) selective infiltration etching (by coating the material with a glass infiltrant and heating it above its glass transition temperature), (4) polishing (with silicon carbide paper and diamond or silica suspension), (5) laser treatment (offering the advantage of no surface contamination), (6) UV-light treatment (which induces superhydrophilicity), (7) coating (using hydroxyapatite or calcium phosphate to enhance surface adhesion), and (8) biofunctionalization (immobilization of biomolecules to improve biochemical properties and biological tissue response) [37,38,39,40,41,42,43]. The diversity and ongoing refinement of Y-TZP surface modification techniques hold promise for achieving superior clinical outcomes in the near future. In addition to surface modification, lasers play an increasingly important role in implant dentistry by enabling effective decontamination of implant surfaces and modulation of cellular responses [44,45,46,47,48], as illustrated in Figure 1.

In recent years, lasers have emerged as versatile tools in implant dentistry, offering unique advantages in surface engineering and biological modulation, as well as serving as effective instruments for debridement in peri-implant diseases [49,50,51,52,53,54,55,56]. Various laser systems—including Er:YAG, Er,Cr:YSGG, Nd:YAG, CO_2_, and diode lasers—have been investigated for their potential to modify zirconia surfaces [57,58,59,60,61]. Erbium-based lasers, owing to their high absorption in water, can effectively modify zirconia surfaces by increasing surface roughness, wettability, and surface energy without inducing chemical contamination [62,63,64]. Such modifications create favorable micro- and nano topographies that enhance fibroblast adhesion, spreading, and collagen fiber orientation, thereby improving soft-tissue attachment and the mucosal seal around implants [22,65,66,67]. Nd:YAG laser irradiation, on the other hand, has been shown to generate controlled microgrooves and nanostructures without compromising the mechanical integrity of zirconia, while promoting gingival fibroblast viability and proliferation [1,26,27,28,68]. Beyond surface modification, lasers are valuable tools for the decontamination of implant surfaces affected by peri-implant disease. Numerous studies have reported significant reductions in biofilm and endotoxin loads while maintaining fibroblast biocompatibility [69,70]. Furthermore, low-level laser therapy (LLLT), through photobiomodulation, has gained attention for its ability to stimulate mitochondrial activity, accelerate fibroblast proliferation, and enhance collagen synthesis, leading to improved wound healing and greater stability of peri-implant soft tissues [71,72,73,74,75]. All together, these findings suggest that laser-based approaches represent powerful strategies to optimize zirconia implant surfaces and promote the health and integration of surrounding soft tissue. Despite the growing clinical interest in zirconia implants, no systematic review has specifically addressed how different surface modifications of zirconia influence fibroblast behavior [17,76,77,78,79]. Most existing reviews focus primarily on osseointegration, leaving the soft-tissue perspective largely underexplored [80,81,82]. This represents a significant gap, as fibroblasts constitute the first cellular line of defense at the implant–mucosa interface and play a crucial role in maintaining peri-implant health [23,25]. The lack of a focused synthesis limits the development of evidence-based guidelines for optimizing zirconia surfaces in relation to soft-tissue biology [12]. While lasers can be used both to modify zirconia surfaces and to modulate fibroblast activity directly through photobiomodulation, the scope of the present review is clearly defined to encompass both categories of light-based applications.

Therefore, the present systematic review aims to evaluate the available evidence on fibroblast responses to zirconia surfaces following various light-based applications including laser irradiation.

## 2. Materials and Methods

### 2.1. Focused Question

This systematic review was designed in accordance with the PICO framework [83] and aimed to address the following research question:

Among fibroblast cultures grown on zirconia surfaces (Population), does laser treatment (Intervention) influence cellular behavior and properties (Outcome) compared with non-laser-treated zirconia surfaces (Comparison)?

### 2.2. Protocol

**Figure 2:**
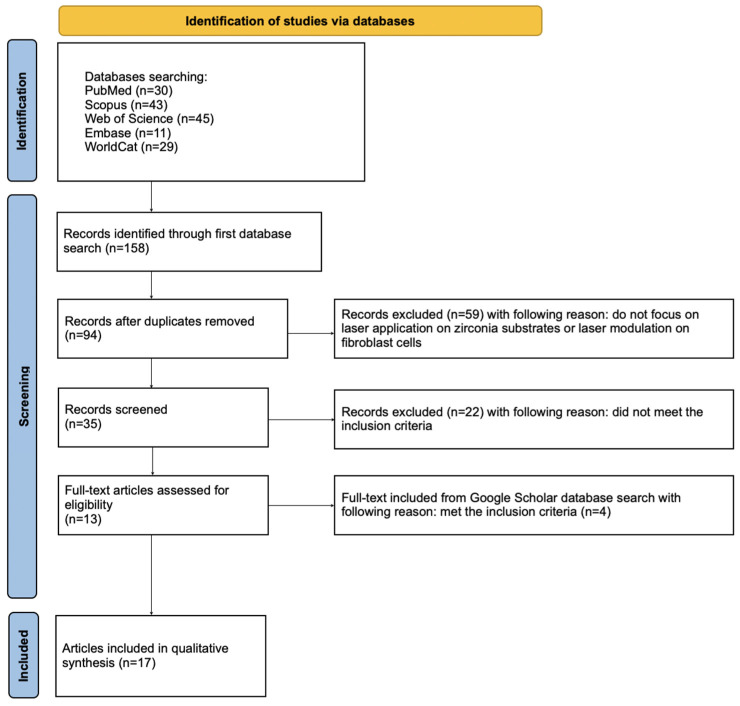
The PRISMA 2020 flow diagram [84].

The article selection procedure was conducted in accordance with the PRISMA guidelines (see Supplementary Materials) [65,84,85,86]. The detailed steps of the selection are illustrated in the flow diagram presented in Figure 2. This review protocol was prospectively registered in the Open Science Framework database and can be accessed at the following link: https://osf.io/4ysvx (accessed on 20 October 2025).

### 2.3. Eligibility Criteria

The following criteria were used to determine the inclusion of studies in the review:

- Application of laser treatment to fibroblasts cultured on zirconia surfaces;
- Experimental in vitro design;
- Presence of a control group;
- Publication written in English.

The exclusion criteria established by the reviewers were as follows:

- Absence of laser application;
- Articles published in languages other than English;
- Randomized controlled trials (RCTs) or non-randomized controlled clinical studies (NRS);
- Clinical case reports;
- Expert opinions or commentaries;
- Editorials;
- Review papers;
- Lack of full-text availability;
- Duplicate publications.

No time restrictions were applied regarding the year of publication.

### 2.4. Information Sources, Search Strategy, and Study Selection

A comprehensive electronic search was carried out in September 2025 across several databases, including PubMed, Scopus, Web of Science (WoS), Embase, and WorldCat. The objective was to identify all relevant in vitro studies evaluating the effects of laser treatment on fibroblast behavior when cultured on zirconia substrates. The search strategy combined controlled vocabulary and free-text terms related to lasers, fibroblasts, and zirconia implants. The general Boolean query used was: (laser) AND (fibroblast) AND (zirconium OR zirconia OR ZrO_2_ OR Y-TZP) AND (implant OR disk OR specimens OR plates OR blocks).

Database-specific search strings were adapted as follows:

- PubMed: (laser[All Fields]) AND (fibroblast[All Fields]) AND (zirconium[All Fields] OR zirconia[All Fields] OR ZrO_2_[All Fields] OR Y-TZP[All Fields]) AND (implant[All Fields] OR disk[All Fields] OR specimens[All Fields] OR plates[All Fields] OR blocks[All Fields])
- Scopus: TITLE-ABS-KEY(laser AND fibroblast AND (zirconium OR zirconia OR ZrO_2_ OR “Y-TZP”) AND (implant OR disk OR specimens OR plates OR blocks))
- Web of Science: TS = (laser AND fibroblast AND (zirconium OR zirconia OR ZrO_2_ OR Y-TZP) AND (implant OR disk OR specimens OR plates OR blocks))
- Embase: (laser:ti,ab AND fibroblast:ti,ab AND (zirconium:ti,ab OR zirconia:ti,ab OR ZrO_2_:ti,ab OR Y-TZP:ti,ab) AND (implant:ti,ab OR disc:ti,ab OR specimens:ti,ab OR plates:ti,ab OR blocks:ti,ab))
- WorldCat: laser AND fibroblast AND (zirconium OR zirconia OR ZrO_2_ OR Y-TZP) AND (implant OR disk OR specimens OR plates OR blocks)

In addition, the reference lists of all eligible studies were manually screened to identify any additional relevant publications. The search and selection process was meticulously executed in accordance with a predetermined set of eligibility criteria, and only studies that were fully available in their entirety were considered in the review.

### 2.5. Data Collection Process and, Data Items

The selection of studies was performed by six independent reviewers (J.K., A.K., M.L., N.S., Z.N., W.S.), who independently assessed each record according to the predefined inclusion and exclusion criteria. For every study that met the eligibility requirements, data were extracted on the first author, year of publication, study design, article title, characteristics of the zirconia surfaces and laser parameters applied, and outcomes related to fibroblast cellular responses. All extracted data were organized and recorded in a structured Excel spreadsheet to ensure consistent and systematic analysis.

### 2.6. Risk of Bias and Quality Assessment

To reduce the potential for selection bias, all reviewers independently screened study titles and abstracts during the initial phase of the review. The degree of inter-rater reliability was calculated using Cohen’s kappa coefficient. In cases where disagreements arose regarding study eligibility, the reviewers convened to discuss each instance, and final decisions were reached through consensus.

### 2.7. Quality Assessment

Two blinded reviewers (J.M. and M.D.) independently evaluated the methodological quality of each included study using the Mixed-Methods Appraisal Tool (MMAT), version 2018. It is a validated instrument that allows for a consistent assessment of various study designs, including randomized, non-randomized, descriptive quantitative, and mixed-methods research. The MMAT includes five key criteria aimed at evaluating methodological rigor, the integration of study components, and the overall validity of the research. The following guiding questions were used in the appraisal process:

- Is the sampling strategy relevant to address the research question?
- Is the sample representative of the target population?
- Are the measurements appropriate?
- Is the risk of nonresponse bias low?
- Is the statistical analysis appropriate to answer the research question?

Each criterion was rated as “yes,” “no,” or “can’t tell.” Any discrepancies between reviewers were resolved through discussion until consensus was achieved. Inter-rater agreement was quantified using Cohen’s kappa coefficient, calculated with MedCalc software (version 23.1.7; MedCalc Software Ltd., Brussels, Belgium). The resulting kappa value of 0.87 (*p* < 0.001) indicated a high level of concordance, reflecting near-perfect agreement between reviewers.

## 3. Results

### 3.1. Study Selection

A comprehensive search of PubMed, Scopus, Web of Science, Embase, and WorldCat initially identified 158 potentially relevant articles. After duplicates’ removal, 35 records remained for title and abstract screening. Additionally, reference lists of the included papers were reviewed to identify any further eligible studies. Only in vitro and animal studies were included, as these models enable controlled assessment of fibroblast-specific outcomes, such as adhesion or viability, following laser modification of zirconia surfaces or cell modulation. Clinical studies (RCTs and NRS) were excluded because they typically report broader soft-tissue parameters rather than direct fibroblast behavior and therefore do not align with the mechanistic focus of this review. In total, 17 studies met the inclusion criteria and were incorporated into the qualitative synthesis of this review [87,88,89,90,91,92,93,94,95,96,97,98,99,100,101,102,103].

### 3.2. General Characteristics of the Included Studies

All included studies were performed under in vitro conditions [87,88,89,90,91,92,93,94,95,96,97,98,99,100,101,102,103], with two also incorporating in vivo experiments in rats [93,99]. The majority investigated yttria-stabilized zirconia (Y-TZP) disks [87,88,89,90,91,92,93,94,96,97,99,100,101,102,103], while other material types such as thin films [95], nanocomposite coatings [98], and magnesia-partially stabilized zirconia [96] were also examined.

A wide spectrum of laser systems was used for zirconia surface modification, including Er:YAG [87], Nd:YAG [88,100,102,103], Er,Cr:YSGG [90], diode [91,92], femtosecond [89,93,98,99,101], excimer [95,97], CO_2_ [96], and two-photon excitation lasers [94]. These differed substantially in wavelength, energy density, and exposure time, leading to diverse effects on surface topography and cellular behavior.

Across the studies, Er:YAG irradiation produced micro-roughened, biocompatible surfaces that maintained fibroblast morphology and reduced biofilm formation [87]. Nd:YAG laser treatment supported fibroblast adhesion in some studies [88], while in Fernandes et al. [102] the fibroblast response depended on the presence of an MTA coating, with higher viability observed on MTA-treated zirconia. The Er,Cr:YSGG laser enhanced fibroblast elongation and filopodia formation while demonstrating high antibacterial efficacy against *E. coli* [90]. Diode laser irradiation, particularly at 3 J/cm^2^, improved fibroblast proliferation, organization, and IL-6/IL-8 secretion, confirming its stimulatory effect [91,92].

Excimer lasers increased vinculin, integrin β_1_, and collagen I expression, indicating stronger adhesion and cytoskeletal organization [97]. Femtosecond laser–treated surfaces consistently promoted fibroblast alignment, migration, and proliferation, with additional in vivo confirmation of improved soft-tissue healing and reduced inflammation [89,93,98,99,101]. CO_2_ laser modification of magnesia-stabilized zirconia improved fibroblast adhesion at optimal power densities (1.6 kW/cm^2^) without compromising cell viability [96]. Finally, two-photon laser excitation combined with fluorescence-lifetime imaging provided non-invasive monitoring of fibroblast metabolism, demonstrating high cellular viability and biocompatibility of zirconia surfaces [94].

Human gingival fibroblasts were the most frequently used cell type, while several studies utilized mouse fibroblast lines [90,94,95,97,98]. Hao et al. [96] and Gnilitskyi et al. [99] investigated human dermal fibroblasts. Most experiments evaluated fibroblast adhesion, morphology, proliferation, and viability [87,88,90,91,92,93,94,95,96,98,100,101,102,103], while some assessed cytokine secretion, gene expression, or cytotoxicity [89,91,95,97]. In addition, selected studies examined osteoblast responses [88,100], biofilm formation [87], or tissue integration in vivo [99].

The general characteristics of the included studies and the corresponding laser parameters are summarized in Table 1.

### 3.3. Main Study Outcomes

This review analyzed fibroblast responses to laser-modified zirconia surfaces, focusing on adhesion, morphology, proliferation, viability, gene expression, and cytokine secretion. Overall, the evidence demonstrates that laser surface modification of zirconia is biocompatible and, in several cases, promotes favorable fibroblast attachment and organization. The extent of these effects depends on the zirconia composition, the applied laser parameters, and the type of experimental model. A comprehensive summary of the analyzed variables and study findings is presented in Table 2.

#### 3.3.1. Surface Characteristics

Laser treatment consistently altered zirconia surface topography, with specific effects depending on the laser type and irradiation parameters. Er:YAG irradiation [87] produced micro-roughened surfaces without inducing thermal damage, whereas Er,Cr:YSGG treatment [90] modified the surface morphology while preserving overall structural integrity. Nd:YAG laser processing resulted in localized surface melting or microcrack formation, although this effect was reported only in one study using high-energy settings [88]. CO_2_ laser modification of magnesia-stabilized zirconia generated a roughened microstructure that promoted improved cell attachment [96].

Femtosecond laser irradiation produced well-defined micro and nanostructures, including periodic surface patterns and laser-induced periodic surface structures (LIPSS), which markedly modified topography and increased surface complexity [89,93,98,99,101]. These surface patterns were closely associated with enhanced fibroblast attachment and alignment reported in multiple studies [89,92,93,97,98,99].

#### 3.3.2. Fibroblast Adhesion and Morphology

Most studies reported improved fibroblast adhesion, spreading, and cytoskeletal organization following laser modification of zirconia surfaces. Er:YAG irradiation supported normal cell morphology and stable attachment without inducing cytotoxicity [87]. CO_2_-treated magnesia-stabilized zirconia surfaces also enabled clear fibroblast adhesion, with cells exhibiting a flattened morphology consistent with mature attachment [96]. Femtosecond laser–generated micro- and nanostructures promoted pronounced cytoskeletal organization, including elongated cell bodies, alignment along surface patterns, and well-defined spreading on laser-textured regions [89,93,98,99,101]. Excimer laser treatment enhanced fibroblast adhesion primarily through increased hydrophilicity and focal adhesion formation, with elevated vinculin localization and more prominent filopodia reported specifically in the study by Akashi et al. [97]. In contrast, fibroblast behavior on PLD-derived thin films (Jelínek et al. [95]) remained normal but without the extensive filopodia or focal adhesion markers observed in [97]. Er,Cr:YSGG irradiation resulted in elongated, spindle-shaped fibroblasts with abundant filopodia, indicating enhanced migratory and adhesive capacity [90]. Photobiomodulation with a diode laser improved fibroblast morphology and cytoskeletal organization on zirconia surfaces, particularly at moderate energy densities [91], whereas the diode microlaser used for instrumentation in Lang et al. [92] did not produce measurable differences in adhesion or morphology. Finally, da Cruz et al. [103] reported no significant differences in fibroblast adhesion or viability between Nd:YAG-treated zirconia and conventionally prepared surfaces, indicating that laser microgrooves in that protocol did not further enhance cell attachment. Despite such isolated findings, the majority of studies demonstrate that laser-induced micro- and nanostructuring enhances fibroblast adhesion and promotes more advanced cytoskeletal development.

#### 3.3.3. Proliferation and Viability

Across the included studies, fibroblast proliferation and viability were generally maintained or improved following laser treatment, regardless of the laser system applied. Er:YAG irradiation supported normal fibroblast viability and did not induce cytotoxicity [87]. Photobiomodulation with a diode laser enhanced fibroblast viability in a dose-dependent manner, with the 3 J/cm^2^ protocol producing the highest cell counts and metabolic activity [91]. In contrast, the diode microlaser used for instrumentation did not alter fibroblast viability compared with untreated zirconia [92]. Femtosecond laser–treated surfaces supported favorable fibroblast proliferation and metabolic activity, with several studies reporting increased cell numbers and enhanced spreading over time [89,93,98,99,101]. Nd:YAG-treated surfaces generally sustained normal fibroblast viability and proliferation, although the study by da Cruz et al. [103] reported no significant differences between laser-treated and conventionally finished surfaces, indicating that Nd:YAG microgrooves did not further stimulate fibroblast growth under those conditions. CO_2_-treated zirconia surfaces allowed fibroblasts to attach and remain viable, although explicit quantitative proliferation data were not provided [96]. Excimer laser-treated zirconia similarly supported fibroblast viability and normal proliferation dynamics, with no evidence of cytotoxic effects [97]. Importantly, none of the included studies reported a reduction in fibroblast viability after laser application, confirming the overall biocompatibility of all evaluated laser protocols.

#### 3.3.4. Gene Expression and Cytokine Secretion

Laser treatments influenced fibroblast cytokine and gene expression profiles in a manner that depended strongly on the laser parameters and cell model used. Photobiomodulation with a diode laser significantly modulated inflammatory mediators, with IL-6 and IL-8 levels increasing in a dose-dependent manner, while VEGF gene expression remained largely unchanged [91]. Femtosecond-structured zirconia surfaces were associated with reduced inflammatory cytokine expression and promoted a shift toward an anti-inflammatory environment, as observed particularly in studies evaluating fibroblast behavior under inflammatory conditions [93,99]. Excimer laser irradiation increased the expression of adhesion-related genes, including integrin β1 and collagen I, indicating enhanced focal adhesion formation and early extracellular matrix synthesis [97]. In contrast, Nd:YAG-treated surfaces did not significantly alter IL-8 secretion in fibroblasts compared with control conditions [103]. The study by Marques et al. reported stable IL-6 levels over time across all surface types, without laser-specific up- or down-regulation [100]. Importantly, Er:YAG and several other laser systems did not assess fibroblast cytokine or gene expression directly [87,90,92,96,98,101], and therefore no conclusions can be drawn for these modalities.

#### 3.3.5. Additional Findings

Several studies also evaluated additional biological and microbiological outcomes beyond fibroblast behavior. Er:YAG [87] and Er,Cr:YSGG [90] lasers demonstrated antibacterial activity, effectively reducing bacterial load or biofilm accumulation compared with mechanical or chemical decontamination methods. Nd:YAG-treated zirconia surfaces showed a more pronounced influence on osteoblast function than on fibroblasts in several studies, particularly with regard to osteocalcin and inflammatory mediator expression [100,102], while no enhancement of fibroblast viability or proliferation was observed in da Cruz et al. [103]. Femtosecond laser–patterned zirconia surfaces promoted improved soft-tissue integration in vivo, including reduced inflammation and lower neutrophil infiltration in animal models [93,99]. In contrast, femtosecond laser treatment in Aivazi et al. [101] was limited to in vitro fibroblast assays and did not include in vivo evaluation.

### 3.4. Quality Assessment of Individual Studies

For all of the five MMAT questions, two papers received a positive answer to all five of them [93,99], while fifteen papers received a positive answer to four questions [87,88,89,90,91,92,94,95,96,97,98,100,101,102,103] (see Table 3).

## 4. Discussion

This review aimed to evaluate and synthesize the available evidence on the effects of light-based surface treatments—particularly laser irradiation—on fibroblast responses to zirconia. Given that the studies included different types of lasers (Er:YAG, Nd:YAG, CO_2_, diode) with different wavelengths and tissue interaction properties, direct comparisons of results should be interpreted with caution. Nevertheless, certain patterns emerge that are worth discussing. Notably, da Cruz et al. [103] demonstrated that Nd:YAG laser microgrooves alone (45–125 µm width, 20–50 µm depth) on sandblasted/acid-etched zirconia did not significantly alter fibroblast viability, proliferation, or collagen production compared to conventional surface treatment, suggesting that not all laser modifications provide biological advantages beyond standard surface preparation. Overall, most included studies were consistent with the evidence synthesized in this review, showing enhanced fibroblast adhesion, proliferation, and cytoskeletal organization on laser-treated zirconia, indicating improved biological compatibility [87,88,89,90,91,92,93,94,95,96,97,98,99,100,101,102,103]. These outcomes align with the widely reported influence of zirconia surface physicochemistry—especially its hydrophilicity and surface energy—plays a critical role in early protein adsorption and subsequent soft-tissue attachment [104,105,106], in agreement with the findings of Tang et al. [23].

Likewise, previous studies confirm that fibroblast adhesion depends strongly on surface chemistry and wettability [104,107], and that photonic treatments such as laser or UV irradiation can activate the surface to promote a more favorable soft-tissue response [1,23,26,27,28,108]. However, not all studies were fully concordant. While Akashi et al. [97] demonstrated that Xe excimer laser treatment increased surface wettability and superhydrophilicity without altering roughness—results that align with the generally positive effects observed in this review—da Cruz et al. [88] reported localized melting and microcrack formation after Nd:YAG irradiation. These microstructural changes may compromise mechanical integrity [109,110,111] and negatively influence osteoblast behavior, even though fibroblast viability remained unaffected. In contrast, femtosecond laser processing [98,99] produced well-defined laser-induced periodic surface structures (LIPSS) that guided fibroblast alignment and enhanced adhesion, fully supporting the evidence that nanoscale structuring improves fibroblast organization and attachment [107,112,113,114]. The evidence indicates that while laser-induced nanostructuring generally enhances zirconia’s biological performance by increasing surface energy and topographical organization [114,115,116,117,118], the magnitude and consistency of these effects depend on precise control of wavelength and energy density [119,120,121,122]. Deviations from optimal parameters may lead to unwanted thermal alterations, whereas controlled nanostructuring appears to create the most favorable conditions for fibroblast attachment and long-term soft-tissue integration [119,120,123,124]. Laser irradiation produced variable effects on fibroblast proliferation, which depended strongly on both the applied energy settings and the experimental design [65,119,125,126].

Several studies were consistent with the synthesized evidence, showing that moderate-energy irradiation enhanced fibroblast growth and organization. Stein et al. [87] reported high fibroblast viability on Er:YAG-treated zirconia compared with chemically decontaminated surfaces, with minimal cytotoxicity—consistent with the generally favorable cellular responses reported across the included studies. Likewise, Gnilitskyi et al. [99] and Petrović et al. [98] demonstrated that femtosecond laser structuring promoted fibroblast attachment and proliferation through the formation of well-organized micro–nanotopographies, supporting the role of controlled surface patterning in facilitating cell growth. However, other results were less consistent. Da Cruz et al. in both studies [88,103] found no significant differences in fibroblast adhesion or viability between Nd:YAG-treated and untreated zirconia, indicating that excessive energy input or localized melting may limit fibroblast responsiveness. Similarly, Pansani et al. [91] reported that diode laser exposure at 3 J/cm^2^ partially reversed LPS-induced cytotoxicity, restoring fibroblast viability and cytoskeletal organization. This partial recovery, while positive, suggests that the beneficial range of laser parameters is narrow and strongly context-dependent.

The broader biological effects of laser modification on zirconia implant surfaces revealed both consistent and divergent findings across studies, reflecting the influence of different irradiation parameters and experimental designs [127,128,129,130,131]. Several investigations demonstrated that laser conditioning improved surface characteristics and cellular responses. Stein et al. [87] observed a reduction in biofilm formation after Er:YAG laser treatment, confirming effective decontamination while maintaining zirconia surface integrity—consistent with the generally positive biological effects of moderate-energy laser application identified in this review. Similarly, Pham et al. [90] reported antibacterial activity of the Er,Cr:YSGG laser against *E. coli*, supporting the observation that laser processing can provide dual benefits of surface biocompatibility and microbial control. Other studies also aligned with the findings regarding fibroblast behavior. Akashi et al. [97] found that excimer laser irradiation enhanced surface wettability and upregulated integrin β1 and collagen I expression, promoting stronger soft-tissue attachment—consistent with the improved adhesion and proliferation trends summarized in this review. Likewise, Petrović et al. [98] and Gnilitskyi et al. [99] confirmed that femtosecond laser–induced periodic structures enhanced fibroblast spreading and alignment, with Gnilitskyi’s in vivo results further validating improved connective tissue integration and mechanical stability. However, not all findings were fully consistent. Pansani et al. [91] reported increased IL-6 and IL-8 expression after diode laser exposure, suggesting that certain energy levels may transiently stimulate proinflammatory signaling. In contrast, Marques et al. [100] observed no differences among surfaces in IL-1β or IL-6 release- IL-6 levels gradually declined, while IL-1β remained barely detectable across all groups. Although this response contrasts with the generally favorable fibroblast viability observed in most studies, it may represent an adaptive phase of cell activation rather than cytotoxic stress. Similarly, da Cruz et al. [88] noted that Nd:YAG treatment caused microcracks and local melting, which did not impair fibroblast viability but might negatively influence other cell types such as osteoblasts—highlighting the importance of carefully selecting laser parameters.

The present review has certain limitations related to the design and methodology of the available studies on light-based surface modification of zirconia. The analyzed studies exhibited substantial heterogeneity in irradiation parameters—such as wavelength, pulse duration, exposure time, and energy output—which complicates direct comparison and limits the generalizability of the results [87,88,89,90,91,92,93,94,95,96,97,98,99,100,101,102,103]. This variability was also reflected in this review, where differences in energy input and laser type (e.g., Nd:YAG vs. femtosecond) produced divergent fibroblast responses, emphasizing the importance of precise parameter control. Moreover, surface characterization methods varied considerably between studies, and essential physicochemical parameters—including surface roughness, surface energy, and wettability—were often reported inconsistently [87,88,89,90,91,92,93,94,95,96,97,98,99,100,101,102,103]. Such methodological discrepancies reduce the reliability of cross-study comparisons and may explain some of the observed inconsistencies in cellular outcomes. Another important limitation is the exclusive inclusion of in vitro and animal studies. Although these models allow controlled evaluation of fibroblast-specific responses, they cannot fully replicate the complex oral environment or long-term soft-tissue dynamics, which may limit the direct translation of the findings to clinical settings. Additionally, the search strategy, may still have inherent limitations due to variability in indexing terminology, which carries a minimal but present risk of missing relevant studies. Furthermore, most investigations were conducted under simplified conditions and over relatively short observation periods, which may not accurately reproduce dynamic tissue remodeling processes [87,88,89,90,91,92,94,95,96,97,98,100,101,102,103]. These limitations highlight the need for standardized protocols that integrate detailed surface characterization, consistent biological models, and reproducible laser parameters. Future research should also incorporate extended in vivo studies to verify whether the favorable fibroblast behavior observed in vitro translates into improved peri-implant soft-tissue integration and long-term clinical stability. Collectively, these considerations underline the necessity for methodological consistency and longitudinal research to validate the clinical relevance of light-based zirconia surface modifications and to establish evidence-based guidelines for their optimal application.

## 5. Conclusions

Within the limitations of the available evidence, this systematic review indicates that laser surface treatment enhances the biological response of fibroblasts cultured on zirconia surfaces. Most of the included studies reported improved fibroblast adhesion, proliferation, and cytoskeletal organization following laser irradiation, suggesting that controlled photonic modification can effectively tailor zirconia topography and surface chemistry to promote soft-tissue compatibility. The presented findings support the concept that laser-induced micro- and nanostructuring improves fibroblast behavior by increasing surface energy and biointerface activation. However, considerable heterogeneity in study design, irradiation parameters, and surface characterization methods prevents direct comparison across studies and limits the generalizability of outcomes. Future investigations should focus on standardized protocols, quantitative evaluation of surface properties, and long-term in vivo validation to identify optimal laser parameters for achieving durable soft-tissue integration around zirconia implant abutments.

## Figures and Tables

**Table 1:** General characteristics of the included studies.

Authors	Aim of the Study	Material and Methods	Results	Conclusions
Stein et al. [87]	Investigation of the efficiency of different implant decontamination methods regarding biofilm modification and potential cytotoxic effects on titanium and zirconium surfaces.	Titanium (1.3 µm) and zirconium (1.1 µm) disks were coated with a multispecies high-adherence biofilm (6 strains) and decontaminated using curette, ultrasonic scaler, glycine or erythritol powder air-polishing, and Er:YAG laser. Cell studies with immortalized human gingival fibroblasts (hGF-hTERT)—cultured 24–48 h to assess cytocompatibility.	Ultrasonic scaler, glycine powder AP, erythritol AP, Er:YAG reduced biofilm activity with air-polishing methods being most effective. Fibroblast assays presented high viability and low apoptosis rate after mechanical and laser treatment. CHX, PVI and doxycycline and NaOCL caused significant cytotoxicity PVI killed almost all fibroblasts	Air-polishing and ultrasonic scaler were most effective and presented best biological effect. Er:YAG reduced biofilm but less effective than other groups, chemicals presented cytotoxic effect.
da Cruz et al. [88]	Comparison of Nd:YAG laser vs. milling grooves on zirconia for osteoblast and fibroblast response	3Y-TZP disks, grooves prepared by milling or Nd:YAG laser. Cells: hFOB osteoblast, immortalized HGF.	Milling improved osteoblast viability, collagen and osteopontin. In laser group fibroblasts showed little difference.	Milling favored osteoblasts over laser; fibroblasts groups shown no significant difference.
Staehlke et al. [89]	Assessment of physico-chemical modification of the zirconia neck region of implants to improve integration of the implant to surrounding tissue.	Polished zirconia disks were subjected to laser microstructuring, a process that entailed the creation of periodic cavities and convex waves, then activated with argon plasma. Characterization: SEM for surface topography and water contact angle for wettability. Cell studies with HGF-1—morphology, spreading, and actin cytoskeleton assessed during the first 24 h.	The laser-microstructered surfaces (originally hydrophobic), after argon plasma activation became hydrophilic (60 μm cavities 13.7°). HGF-1 cells on laser-induced waves spread well. Argon plasma activation promotes better adhesion and spreading of HGF-1 cells into the cavities.	The combination of laser and argon plasma activation of zirconia has been demonstrated to yield a solution that facilitates optimal gingival cell attachment.
Pham et al. [90]	Evaluation of usage of the laser on the surface roughness of zirconia disks. Assessment of single specie biofilm *E. coli* removal after laser treatment. Analysis of the amount of fibroblast adhesion and proliferation utilizing after laser treatment.	Yttria-stabilized zirconia (Y-TZP) disks (10 mm × 2 mm) were divided into four groups: control, Er,Cr:YSGG laser, ultrasonic scaler, and hand instrument (curette) treated. Characterization: visual inspection, SEM, and profilometry. Bacterial decontamination tested with GFP–*E. coli* using fluorescence microscopy and ImageJ (National Institutes of Health, Bethesda, MD, USA) counts. Cell studies—fibroblast adhesion assessed by CellTiter-Glo assay and SEM. Data analyzed by ANOVA.	Ultrasonic and hand scalers caused surface damage, while Er,Cr:YSGG laser did not alter roughness. Bacterial decontamination was most effective with the laser and ultrasonic treatment. Fibroblast adhesion was highest in control and laser groups, with laser-treated cells showing elongated morphology and filopodia, suggesting enhanced migration and growth.	The Er,Cr:YSGG laser effectively removed bacteria, outperforming hand instrumentation, and promoted stronger fibroblast adhesion.
Pansani et al. [91]	To evaluate whether specific photobiomodulation parameters can enhance metabolism and modulate the inflammatory response of gingival fibroblasts on titanium and zirconia surfaces after exposure to Escherichia coli LPS.	Standardized zirconia disks were polished and sintered. Primary gingival fibroblasts were cultured and irradiated with PBM (0.5, 1.5, 3 J/cm^2^) three times at 24 h intervals, with or without *E. coli* LPS exposure. Cell studies—viability, IL-6 and IL-8 synthesis, IL-6 and VEGF gene expression, and fibroblast morphology evaluated.	Fibroblasts on zirconia disks showed reduced viability after LPS exposure and lower survival under laser compared with titanium. IL-6 synthesis was lower, though gene expression was elevated with LPS and modulated by PBM at 3.0 J/cm^2^. IL-8 increased with LPS and was best regulated by PBM at 1.5 J/cm^2^, while VEGF was not upregulated by PBM. Morphologically, LPS impaired spreading, but PBM at 3.0 J/cm^2^ enhanced proliferation and cytoskeletal organization.	Specific PBM parameters can enhance fibroblast metabolism and modulate inflammation on Ti and ZrO_2_ surfaces, potentially improving soft-tissue attachment and reducing peri-implant inflammation.
Lang et al. [92]	To assess how titanium and plastic instruments, a diode laser, and a titanium brush affect the surface properties and fibroblast adhesion on zirconia disks.	Forty zirconia disks were treated with a plastic or titanium curette, diode laser, or rotary titanium brush, or left untreated. Each disk received 20 or 100 strokes to simulate clinical instrumentation. Characterization: surface roughness by optical profilometry. Cell studies—fibroblast adhesion evaluated by SEM cell counts.	For zirconia disks, instrumentation with plastic or titanium curettes, diode microlaser, or a rotary titanium brush caused only negligible changes in surface roughness. Fibroblast adhesion showed no meaningful variation between the different treatment methods.	For zirconia implants, repeated instrumentation did not alter surface roughness, and plastic curettes supported better fibroblast attachment than titanium curettes.
Sun et al. [93]	Assessment of biological response of human gingival fibroblasts (HGFs) and inflammatory response to micro- nano-structured zirconia surfaces created with a femtosecond laser.	Yttria-stabilized zirconia specimens were femtosecond laser-treated to create microgrooves (G3: 30 µm × 5 µm × 30 µm; G6: 60 µm × 5 µm × 60 µm). Cell studies with HGFs were conducted. Characterization: surface analysis, XRD, flexural strength, protein adsorption, and cell morphology, proliferation, and migration under inflammatory conditions. Gene expression and key gene verification by RNA sequencing. In vivo inflammatory response tested in rats. Statistical analysis performed.	Modified zirconia surfaces: decreased levels of proinflammatory cytokines and increased levels of anti-inflammatory cytokines, promoted expression of adhesion related molecules, promoted adhesion of HGFs (well developed cells arranged in the direction of microgrooves), decreased neutrophil infiltration and increased M2-type macrophage polarization.	Prepared surfaces exhibited lower inflammatory response and higher HGFs adhesion. Group with narrower groves (30 µm) performed better than other experimental group (60 µm) and control (polished).
Lepekhina et al. [94]	The study aimed to assess how ceramic implant surfaces made of zirconia influence fibroblast metabolic activity using laser-based imaging.	Mouse 3T3 fibroblasts were cultured on Y-TZP and ATZ zirconia ceramics. Characterization: two-photon laser excitation and FLIM imaging of NAD(P)H and FAD. Cell studies—viability by MTT assay and morphology by SEM.	The FLIM analysis showed that fibroblasts on ceramic surfaces shifted towards a glycolytic metabolic state (more free NAD(P)H), suggesting hypoxia. Despite this, MTT tests and SEM confirmed high viability and normal morphology. Y-TZP supported slightly better cell proliferation than ATZ.	Fibroblasts cultured on zirconia-based implants remained viable and metabolically active. Two-photon FLIM imaging revealed a shift toward glycolysis, likely due to surface porosity, indicating zirconia supports fibroblast attachment and growth while affecting oxygen-dependent pathways.
Jelínek et al. [95]	The purpose of the study was to investigate how zirconia and hydroxyapatite/zirconia coatings, created using laser-based deposition techniques, affect fibroblast behavior in vitro.	ZrO_2_ and HA/ZrO_2_ thin films were deposited on titanium alloy substrates using ArF and KrF excimer lasers. Characterization: XRD, SEM, and WDX. Cell studies with human and mouse fibroblasts—cytotoxicity, attachment, and spreading assessed in vitro.	The coatings were either amorphous or crystalline depending on laser settings. HA/ZrO_2_ films showed no cytotoxic effects and supported fibroblast adhesion and proliferation. In cell culture tests, fibroblasts formed healthy layers on the laser-processed surfaces, with good attachment and even spreading.	The study concluded that zirconia-based coatings enriched with hydroxyapatite, created via pulsed laser deposition on titanium, exhibit both promising mechanical characteristics and excellent compatibility with fibroblasts, supporting their potential application in biomedical implants requiring effective cellular integration.
Hao et al. [96]	The study aimed to evaluate how CO_2_ laser surface modification of magnesia partially stabilized zirconia (MgO–PSZ) affects its ability to support human skin fibroblast adhesion.	MgO–PSZ ceramic blocks were CO_2_ laser-treated at different power densities. Characterization: surface morphology, roughness, and oxygen content. Cell studies with human skin fibroblasts—adhesion analyzed by SEM after 7 days.	No fibroblasts adhered to untreated zirconia, while CO_2_ laser-treated surfaces showed significant cell attachment. Laser treatment increased surface roughness and oxygen content, both of which correlated with improved fibroblast adhesion. Optimal results were observed at 1.6 kW/cm^2^.	CO_2_ laser treatment effectively modifies the surface of MgO–PSZ, promoting favorable conditions for fibroblast adhesion by increasing roughness, oxygen content, and forming beneficial microstructures.
Akashi et al. [97]	The study aimed to investigate whether excimer laser treatment of zirconia surfaces enhances fibroblast adhesion.	Polished zirconia disks were divided into control and laser-treated groups. Characterization: surface roughness and wettability. Cell studies with L929 fibroblasts—adhesion evaluated by qRT-PCR (integrin β1, collagen I α1), 3D laser microscopy (morphology), and confocal microscopy (vinculin distribution).	Excimer laser treatment did not significantly affect surface roughness but markedly increased hydrophilicity. Fibroblasts on laser-treated zirconia exhibited higher expression of adhesion-related genes, developed elongated filopodia and microspikes, and showed stronger vinculin staining, indicating improved adhesion compared with controls.	The findings suggest that excimer laser irradiation improves fibroblast adhesion to zirconia by enhancing hydrophilicity and promoting focal adhesion formation.
Petrović et al. [98]	Assessment of novel titanium-zirconium nanocomposite coating modified by ultrafast laser in terms of cell response.	Titanium–zirconium multilayers were produced by ion sputtering and surface-modified using a Yb:KGW laser. Characterization: AES, XPS, optical microscopy, SEM–EDS, AFM, and TEM. Cell studies with NIH 3T3 fibroblasts included SEM analysis after 2 and 4 days.	Laser-processed surfaces featured uniformly distributed LIPSS (4–7 µm) with increased roughness (Ra = 65 nm) and total surface area. Laser modification enhanced chemical reactivity, forming a carbon-rich oxide layer. Fibroblast adhesion and proliferation were evident by 2 days, with cells aligning along LIPSS orientation and increased growth at 4 days, indicating faster proliferation on the modified surface.	High regularity LIPSS achieved with laser parameters: a pulse energy of 2.5 µJ and scan velocity of 3 mm s-1 Laser modification helped to achieve desired composition changes for optimal biocompatibility.
Gnilitskyi et al. [99]	Biocompatibility assessment of femtosecond laser nanotexturised surfaces of Ti6Al4V and Zr implants	Highly regular LIPSS were formed on polished Ti6Al4V and Zr surfaces. Characterization: SEM, FIB, AFM, surface roughness, XPS. Contact angle was measured. HDFa cell culture tested via Alamar Blue assay (viability). Animal study: samples implanted in male rats.	HR-LIPSS showed high regularity and homogeneity under SEM, greater on titanium than zirconium. Metal surfaces were highly oxidized. Cell attachment and viability were similar on Ti and Zr, with higher attachment on modified versus polished surfaces. In vivo, modified samples were covered with connective fibers and cells by 10 days, achieving full integration by 30 days.	Femtosecond laser generated HR-LIPSS improve cell adhesion and proliferation. Evidence suggests that surface topography has predominant role in cell proliferation but composition also plays a role on biological processes.
Marques et al. [100]	Assessment of osteoblasts and gingival fibroblasts response to Nd:YAG laser textured zirconia surfaces compared to conventionally treated surfaces.	Thirty six zirconia disks were prepared and treated either with two Nd:YAG laser parameters or sandblasting and acid-etching. Characterization: surface roughness and wettability Cell study: with human osteoblasts and fibroblasts cell viability measured after 1, 3 and 7 days, cytokine release and cell morphology and adhesion assessed using ELISA, fluorescence microscopy, and FEG-SEM measured on day 1 and 3.	Osteoblasts showed comparable survival on all surface types, while fibroblasts grew better on laser-treated zirconia than on the sandblasted and acid-etched disks. Inflammatory cytokine levels were similar among groups, and imaging demonstrated normal cell shape, early attachment, and a higher presence of fibroblasts on the laser-modified surfaces.	Osteoblasts respond similarly to laser-textured and conventionally treated zirconia, while fibroblasts perform even better on the laser-modified surfaces.
Aivazi et al. [101]	Comparison of the influence of two zirconia surface modifications- laser treatment and hydroxyapatite-zirconia nanocomposite coating on L929 fibroblast cells viability.	Three groups of A-Y-TZP20 zirconia nanocomposite disks were prepared: untreated, laser-treated with microgrooves, or laser-patterned and then coated with a hydroxyapatite–zirconia nanocomposite. Cell study: L929 fibroblast cells- cell morphology and cell viability was assessed used SEM and MTT assay.	Both surfaces supported cell growth, but laser-treated surfaces, especially the laser-treated surface with hydroxyapatite–zirconia nanocomposite coating, showed higher cell viability and proliferation than the untreated surface, comparable to the control.	Laser surface treatment improves L929 cell viability and proliferation, making treated surfaces more biocompatible than untreated ones.
Fernandes et al. [102]	To evaluate a novel surface treatment—Nd:YAG laser texturing with MTA coating on zirconia implant.	4 groups were tested: MTA-coated laser-textured zirconia (Zr MTA), laser-textured zirconia (Zr textured), polished zirconia (Zr), and polished titanium (Ti). Human osteoblasts (hFOB 1.19) and gingival fibroblasts (HGF hTERT) were cultured for 1–14 days. Cell viability, morphology, osteocalcin, and IL-8 secretion were measured. *Streptococcus oralis* CFUs were counted at 24 h to assess antibacterial activity.	Zr textured showed significantly higher roughness (Ra = 27.73 ± 3.22 µm) vs. other groups. Osteoblast viability was lower on Zr MTA at all timepoints, but these cells showed enhanced differentiation with higher osteocalcin levels at day 3 and increased IL-8 secretion. Fibroblast viability was higher on Zr MTA vs. Zr textured at days 3 and 7.	MTA coating on laser-textured zirconia promoted osteoblast differentiation and fibroblast proliferation but did not demonstrate antibacterial effect against *S. oralis*.
da Cruz et al. [103]	Evaluation of the effect of Nd:YAG laser on zirconia surfaces on the behavior of osteoblasts and fibroblasts in comparison with standard surfaces.	60 zirconia disks (3Y-TZP) divided into 4 groups (*n* = 15). Human osteoblasts (hFOB 1.19) and fibroblasts (HGF hTERT) cultured 1–14 days. Measured: viability, proliferation, morphology (SEM, 24 h), ALP (days 7,14), IL-1β (days 1,3), collagen I (days 3,7), osteopontin (days 3,7), IL-8 (days 1,3).	SEM showed cellular adhesion at 24 h with similar morphology across groups. Cells inside grooves were more rounded, between grooves more spread. No differences between osteoblast/fibroblast viability, proliferation, ALP activity, IL-1β, collagen I, osteopontin, or IL-8.	Nd:YAG laser microgrooves (45–125 µm width, 20–50 µm depth) on sandblasted/acid-etched zirconia do not significantly improve cell viability, proliferation, or differentiation compared to conventional surface treatment alone.

CHX—chlorhexidine; PVI—povidone-iodine; SEM—scanning electron microscopy; SEM-EDS—scanning electron microscopy with energy-dispersive X-ray spectroscopy; FEG-SEM—field emission gun scanning electron microscopy; Y-TZP—yttria-stabilized tetragonal zirconia polycrystal; GFP—green fluorescent protein; PBM—photobiomodulation; LPS—lipopolysaccharide; VEGF—vascular endothelial growth factor; XRD—X-ray diffraction; ATZ—aluminum oxide; MTT—3-(4,5-di methyl thiazol-2-yl)-2,5-diphenyltetrazolium bromide; WDX—wavelength dispersive X-ray analysis; AES—Auger electron spectroscopy; AFM—atomic force microscopy; TEM—transmission electron microscopy; XPS—X-ray photoelectron spectroscopy; FIB—focused ion beam technique; MTA- mineral trioxide aggregate.

**Table 2:** Detailed characteristics of the included studies.

Authors	Type of Study	Zirconia Material	Laser Application	Cell Type	Fibroblast Outcomes	Other Biological Outcomes
Stein et al. [87]	In vitro	Zirconia disks (yttria-stabilized zirconium with roughness average of 1.1 μm)	Er:YAG, 100 mJ, 5 W, 50 Hz, 20 s with 0.9% NaCl cooling	Immortalized human gingival fibroblasts (hGF-hTERT)	Adhesion: Normal fibroblast shape (24 h) Proliferation: ↑ mech., air-polish, laser; ↓ CHX, PVI, doxy (48 h) Cytotoxicity: Low laser (6–7%), US, GPAP, EPAP; high CHX (Ti 41%, Zr 27%) (24 h) Apoptosis: High doxy, moderate NaOCl, minimal mech., laser (24 h)	Biofilm: ↓ after US, GPAP, EPAP, Er:YAG; NaOCl effective only on Ti; CHX, PVI—no reduction. XPS: Surface re-exposed after US, GPAP, EPAP; none after Er:YAG.
da Cruz et al. [88]	In vitro	3Y-TZP disks Control group: sand blasted and acid etched Test groups: Nd:YAG laser grooves Milling grooves Test groups were also sandblasted and acid etched	Nd:YAG laser (Sisma, Italy): 6 W, 2000 mm/s, 1064 nm, 20 kHz, 0.3 mJ/pulse, focal spot of 30 µm	Human gingival fibroblasts (immortalized) Human fetal osteoblasts	Viability: Similar across all surfaces (1, 3, 7 d) Adhesion/Morphology: Normal adhesion and morphology (24 h) Collagen I: ↑ after laser vs. SBAE; similar to mechanical (3 d) IL-8: no significant differences (1, 3 d)	Osteoblasts: Better on mechanical vs. laser-grooved surfaces Surface: Nd:YAG caused local melting/microcracks, impairing osteoblast response Conclusion: Fibroblasts less sensitive to surface changes; Nd:YAG did not improve response vs. SBAE or mechanical grooves
Staehlke et al. [89]	In vitro	Yttria-stabilized zirconia disks (diameter: 12 mm, thickness: 1.5 mm)	TruMicro2030 (Yb:YAG laser) - pulse duration: <400 fs - wavelength of 1030 nm - raw beam diameter ~5 mm, focused diameter: 16 μm - feed speed: 1000 mm/s. - fluence: 3.37 J/cm^2^ Scanner-based process strategy was used.	human gingival fibroblasts (HGF-1)	After 48 h: - pronounced actin cytoskeleton network with long fibers throughout the cell - pronounced vinculin contacts (adaptor proteins). HGF-1 did not organize tight junctions in cell–cell contacts. HGF-1 expressed high glycosaminglycan hyaluronal production.	no data
Pham et al. [90]	In vitro	Yttria-stabilized zirconia (YTZP) disks (diameter: 10 mm, thickness 2 mm) sintered and polished.	Er,Cr:YSGG laser(Waterlase iPlus)-short pulse ‘’H’’ mode: - wavelength 2780 nm in -pulse rate 30 Hz - spot size 1 mm - power setting 1.5 W with air/water of 40%/50% s slowly - distance between the tip and the surface about 0.5 mm.	Mouse fibroblasts NIH3T3	Morphology: More elongated, spindle-shaped fibroblasts with filopodia on laser surfaces Cell Count: Higher in laser group Attachment: No significant difference vs. control	Decontamination: Er,Cr:YSGG most effective vs. E. *coli*; no significant difference vs. ultrasound
Pansani et al. [91]	In vitro	ZrO_2_ disks (8 mm diameter, 2 mm thickness) n = 19	Diode laser (InGaAsP) Wavelength: 780± 3 nm Distance: 2.5 cm Power: 0.025 W Energy densities: 0.5, 1.5, and 3 J/cm^2^	Human gingival fibroblasts	Viability: ↓ on ZrO_2_ + LPS; further ↓ with laser (~½ of Ti); highest in 3 J/cm^2^ ZrO_2_ group Morphology: LPS impaired spreading; 3 J/cm^2^ laser improved cell number, spreading, cytoskeleton IL-6 (protein): ↑ in all; lowest in 1.5 J/cm^2^ + LPS; highest in 1.5 J/cm^2^ alone IL-8: ↑ with LPS; better modulated by 1.5 J/cm^2^ laser IL-6 (gene): ↑ with LPS; ↓ after 3 J/cm^2^ laser VEGF (gene): No change after laser vs. control	no data
Lang et al. [92]	In vitro	ZrO_2_ disks 5 mm in diameter) n = 40	Diode laser Time= 60 s Power: 1.4 W Laser mode: continuous mode	Human gingival fibroblasts	Cell count: The highest cell count was observed on zirconia disk surface treated with microlaser, while the lowest cell count was observed on disks treated with titanium curettes especially on the sample with 20 strokes	no data
Sun et al. [93]	In vitro + in vivo (Sprague Dawley rats)	Yttria-stabilized zirconia disks (diameter 15 mm, thickness 1.4 mm)	Ti:Sapphire femtosecond laser system central wave length: 800 nm repetition rate: 1 kHz pulse duration: 38 fs pulse energy: 80 μJ scanning speed: 1.48 mm/s	human gingival fibroblasts (HGF)	Protein Expression: ↑ adhesion-related proteins in G3 (narrow grooves) Morphology: Regular, elongated HGFs with filopodia; best in G3 (24 h) Migration: ↑ in G3 (12 h); G3 & G6 > control (24 h) Proliferation: Similar at 24 h; ↑ in G3 & G6 at 72 h	Cytokines: ↓ proinflammatory, ↑ anti-inflammatory under inflammation; best in G3 Neutrophils: ↓ infiltration in experimental groups; lowest in G3
Lepekhina et al. [94]	In vitro	- ATZ - Y-TZP Cylindrical samples, 10 mm diameter × 5 mm height. Number of samples: Control 10; ATZ 5; Y-TZP 5	Two-photon laser Power: 7 mW Wavelength: 760 nm (NAD(P)H), 830 nm (FAD) Image size: 100 × 100 μm^2^ Resolution: 128 × 128 px Acquisition time: 12 s Fields: 5 per sample	Mouse 3T3 fibroblasts	Adhesion/Morphology: Fibroblasts attached, spindle-shaped with lamellipodia on both zirconia Viability/Proliferation: 24 h—50% (ATZ), 108% (Y-TZP); ↑ at 48 h Metabolism: Shift to glycolysis (↑ free NAD(P)H, shorter lifetimes); FAD/NAD(P)H trend ↑, not significant	Cytotoxicity: None observed Redox Ratio: Slightly ↑ FAD/NAD(P)H on ATZ & Y-TZP vs. control; not significant Zirconia Structure: Porosity may cause mild hypoxia due to reduced oxygen diffusion
Jelínek et al. [95]	In vitro	Zirconia thin films (ZrO_2_) and HA/ZrO_2_ composites on Ti6Al4V disks. Substrates: Ti6Al4V, diameter 10–12 mm, thickness 2 mm. Buffer layer ZrO_2_ ~100 nm.	Technique: Pulsed Laser Deposition (PLD) with excimer lasers (for coatings, not fibroblasts) ZrO_2_ Films: KrF laser, 248 nm, 10 Hz, 450 mJ, ~4 J/cm^2^, 4 cm distance, 20–700 °C, vacuum, ~4000 pulses (~100 nm) HA on ZrO_2_ (Set 1): ArF laser, 193 nm, 50 Hz, 330 mJ, ~6 J/cm^2^, 3 cm, 600 °C, H_2_O vapor 50 Pa, on ZrO_2_/Ti6Al4V HA on ZrO_2_ (Set 2): KrF laser, 248 nm, ~3 J/cm^2^, 6 cm, 600 °C, Ar + H_2_O 40 Pa (Ar/H_2_O ≈ 0.8), on ZrO_2_/Ti6Al4V	Mouse fibroblasts. Human fibroblasts.	Cytotoxicity: HA/ZrO_2_ highly biocompatible; fibroblasts viable Adhesion (24 h): 53% on HA/ZrO_2_, 47% on dish; total similar to control Spreading (96 h): 47% on surface, 53% surrounding; total cell number ~control Morphology: Flattened, evenly distributed cells forming sub- and confluent layers ECM Marker: Homogeneous fibronectin expression	Inflammation: Not reported Osteoblasts: Not reported In vivo Integration: Not investigated Cytotoxicity Controls: HA/ZrO_2_ showed highest fibroblast survival (~94% human, ~78% mouse) compared to red rubber, dental resin, ceramics
Hao et al. [96]	In vitro	Magnesia partially stabilized zirconia (MgO–PSZ) Blocks: 50 × 12 × 2.15 mm. Exact number of samples not specified.	CO_2_ Laser Mode: continuous wave, Power: 3 kW Wavelength: 10.6 μm Spot size: 11 mm (defocused beam) Scanning speed: 2000 mm/min Power densities: 0.5–2.5 kW/cm^2^ Protocol: single pass across the specimen surface Assist gas: oxygen (O_2_), 2 bar	Human skin fibroblasts	Adhesion: None on untreated MgO–PSZ; present on CO_2_ laser-treated surfaces Optimal Laser: 1.6 kW/cm^2^—sharp ↑ in attachment; no further increase at 1.9 kW/cm^2^ Morphology: Flattened fibroblasts reaching final adhesion stage on treated surfaces	Osteoblasts: Not reported Inflammation: Not reported Note: Higher oxygen content and rougher surfaces may enhance future osteoblast integration
Akashi et al. [97]	In vitro	zirconia disks—13 mm diameter, 0.5 mm thickness	Excimer laser (Xe excimer UV lamp) Wavelength: 172 nm Energy: 7.21 eV Total irradiance: 20 mW/cm^2^ Treatment duration: 10 min	L929 fibroblasts (mouse fibroblast cell line)	Surface: Roughness unchanged (0.0078→0.0080 μm); contact angle ↓ 55.3° → 33.1° Gene Expression: Integrin β1 ↑ 1.3×, Collagen I α1 ↑ 1.2× (6 h & 24 h) Morphology: Microspikes ↑ at 3 h, filopodia at 24 h; vinculin ↑ with focal adhesion localization	Adhesion: Excimer laser ↑ zirconia–fibroblast attachment Surface: Superhydrophilicity improved Clinical Potential: Better biological seal may help prevent peri-implantitis
Petrović et al. [98]	In vitro	titanium/zirconium multilayer thin films on silicon substrate Structure: 15 bilayers of Ti/Zr, total thickness 500 nm, individual Ti and Zr layers ~17 nm each	Femtosecond laser - wavelength: 1030 nm - pulse duration: 160 fs - repetition rate: 1 kHz - pulse energy: 2.5 μJ - spot diameter: 43 μm - fluence: 0.662 J/cm^2^ - polarization: Linearly p-polarized Protocol: - scan velocity: 3 mm/s - surface area: 5 × 5 mm - line spacing: 15 μm	NIH 3T3 fibroblasts (established adherent mouse fibroblast cell line)	Surface: Roughness Ra = 65 nm; LIPSS periodicity ~880 nm, depth up to 200 nm Cell Response: ↑ adhesion & proliferation; 2 d—elongated cells along LIPSS (±15°), 4 d—full coverage, longer cells (±30°) Growth: Faster than flat controls, aligned with laser topography	Surface: Biocompatible Ti and Zr oxides; no silicon exposure Cell Response: ↑ adhesion and immobilization; enhanced interactions via increased surface area and topography Guidance: LIPSS direct cell orientation
Gnilitskyi et al. [99]	In vitro and in vivo	In vitro: Ti6Al4V (grade-5 titanium alloy) and Zr (99.7% purity) In vivo: Two groups of rats: Group 1: Titanium alloy and Zirconia with smooth surface implants Group 2: Titanium alloy and Zirconia with femtosecond laser modified surface;	Femtosecond laser - wavelength: 1030 nm - pulse duration: 213 fs - repetition rate: 600 kHz - power: up to 20 W - spot diameter: 10.4 μm (at 1/e2 intensity) - fluence: 0.6 J/cm^2^ (Ti6Al4V), 0.49 J/cm^2^ (Zr) Protocol: - Scanning speed: 3 m/s - Step size: 4 μm	In vitro: HDFa cells (Human Dermal Fibroblasts-Adult) In vivo: Rat cells and fibers covering implants	In vitro: Surface: Roughness ↑ (Ra 0.131 μm Ti6Al4V, 0.148 μm Zr); LIPSS periodicity ~820 nm Ti, 800 nm Zr; contact angles unchanged Cell Response: 2× higher attachment at day 3 vs. controls; sustained 2× proliferation through days 7–10 Comparison: No difference between Ti6Al4V and Zr In vivo: Smooth control Ti and Zr implants showed almost no cell or fiber attachment at 10 and 30 days. All modified implants were fully covered by connective fibers and cells at 10 days. Fibroblasts showed slightly higher density on Zr than on Ti.	In vivo: Cell Density (10 d): Erythrocytes 11.5–13.3/mm^2^, Leukocytes 2.1–3.6/mm^2^, Fibroblasts 7.4–8.8/mm^2^ 30 Days: Full tissue integration, extensive connective tissue, difficult removal
Marques et al. [100]	In vitro	Yttria-stabilized zirconia disks (10 mm of diameter, 2.5 mm of thickness)	Nd:YAG laser - total output power: 6 W - -wavelength: 1064 nm - spot size: 300 μm - pulse energy: 0.3 mJ/Pulse Protocol: - two patterns: Group B: laser 10 μm spacing 10% power (0.6 W), Group C: laser 20 μm spacing 1% power (0.06 W)	Human fetal osteoblasts—hFOB 1.19; Immortalized human gingival fibroblasts (HGF-hTERT)	Cell response: Fibroblast viability rose over time, with laser-treated surfaces outperforming sandblast/acid-etch. Microscopy showed normal cell morphology in all groups, but firmer attachment and pronounced filopodia on laser-textured zirconia.	No differences were detected among surfaces for IL-1β or IL-6 release. IL-6 levels gradually declined, while IL-1β remained barely detectable across all groups, indicating that none of the surface treatments triggered an inflammatory reaction in fibroblasts.
Aivazi et al. [101]	In vitro	A-Y-TZP disks	Femtosecond laser	L929 fibroblasts cells	Cell response: all surfaces supported growth; laser-treated and laser patterned and then coated with a hydroxyapatite–zirconia nanocomposite showed higher proliferation than control; laser-only treated group showed the highest optical density at days 2 and 5	no data
Fernandes et al. [102]	In vitro	Material: Y-TZP Samples: 60 disks total (8 mm diameter, 3 mm thickness) 4 groups: - Zr MTA: Laser-textured + MTA coating - Zr textured: Laser-textured only - Zr: Polished zirconia - Ti: Polished titanium (control)	Laser: Nd:YAG Wavelength: 1064 nm Power: 40% (2.4 W) Pulse Width ~35 ns Scan Speed: 128 mm/s Focus Distance: 328 mm Post-laser treatment: Sintering at 1500 °C for 2 h, sandblasting (Al_2_O_3_, 250 µm, 30 s), HF acid etching (48%, 30 min) MTA coating: MTA Angelus powder + distilled water, applied with spatula, pressed 3–4 h, set 24 h.	Human Fetal Osteoblasts (hFOB 1.19)—ATCC CRL-11372TM Immortalized Human Gingival Fibroblasts (HGF hTERT)	- Morphology: adherent cells all groups; more prominent bodies on Zr MTA/Zr textured - Viability: Zr MTA > Zr textured at days 3,7; no difference vs. Zr or Ti - IL-8: Zr textured highest: vs. Zr (*p* < 0.05), vs. Ti (p < 0.001) day 1; vs. all groups day 3; decreased day 1→3 - Collagen I: days no differences (*p* > 0.05); ~2200 pg/mL maintained	- Antibacterial: S. oralis CFU (24 h)—no difference in all groups ~106 CFU/mL
da Cruz et al. [103]	In vitro	Material: 3Y-TZP Samples: 60 disks (8 mm diameter, 2 mm thickness) Groups: 4 groups (n = 15 each): - Group A: Width 84.12 ± 5.13 µm, Depth 36.35 ± 4.49 µm - Group B: Width 125.07 ± 5.29 µm, Depth 23.01 ± 3.79 µm - Group C: Width 45.36 ± 2.37 µm, Depth 50.54 ± 2.48 µm - Group D (Control): no laser treatment	Laser: Nd:YAG Wavelength: 1064 nm Power 6 W Pulse Width ~35 ns Repetition Rate 20 kHz Focal Spot ~30 µm Focus Distance: 328 mm Post-laser treatment: Sintering 1500 °C (2 h, 8 °C/min heating/cooling), sandblasting (Al_2_O_3_ 250 µm, 6 bar, 30 s, 12 cm distance), HF acid (48%, 30 min, room temp), ultrasonic cleaning.	Human Fetal Osteoblasts (hFOB 1.19)—ATCC CRL-11372TM Immortalized Human Gingival Fibroblasts (HGF hTERT)	- Cell adhesion and morphology: adherent cells in all groups with similar morphology, more rounded phenotype, group C had elongated bodies, similar to control, control showed greater cell quantity - Viability: increased over time in all groups, similar across all surfaces - proliferation: similar rates regardless of groove dimensions - IL-8 concentration: decreased from day 1→3 in all groups, similar inflammatory response across surfaces - Collagen I Production: ~2200–2300 pg/mL maintained across timepoints, stable production in all surface textures	no data

↑—increase; ↓—decrease; →—from/to.

**Table 3:** Risk of Bias of Individual Studies.

Authors	1. Is the Sampling Strategy Relevant to Address the Research Question?	2. Is the Sample Representative of the Target Population?	3. Are the Measurements Appropriate?	4. Is the Risk of Nonresponse Bias Low?	5. Is the Statistical Analysis Appropriate to Answer the Research Question?
Stein et al. [87]	Yes	No	Yes	Yes	Yes
da Cruz et al. [88]	Yes	No	Yes	Yes	Yes
Staehlke et al. [89]	Yes	No	Yes	Yes	Yes
Pham et al. [90]	Yes	No	Yes	Yes	Yes
Pansani et al. [91]	Yes	No	Yes	Yes	Yes
Lang et al. [92]	Yes	No	Yes	Yes	Yes
Sun et al. [93]	Yes	Yes	Yes	Yes	Yes
Lepekhina et al. [94]	Yes	No	Yes	Yes	Yes
Jelínek et al. [95]	Yes	No	Yes	Yes	Yes
Hao et al. [96]	Yes	No	Yes	Yes	Yes
Akashi et al. [97]	Yes	No	Yes	Yes	Yes
Petrović et al. [98]	Yes	No	Yes	Yes	Yes
Gnilitskyi et al. [99]	Yes	Yes	Yes	Yes	Yes
Marques et al. [100]	Yes	No	Yes	Yes	Yes
Aivazi et al. [101]	Yes	No	Yes	Yes	Yes
Fernandes et al. [102]	Yes	No	Yes	Yes	Yes
da Cruz et al. [103]	Yes	No	Yes	Yes	Yes

## Data Availability

Data supporting the findings of this study are available within the article.

## Supplementary Materials

The following supporting information can be downloaded at: https://www.mdpi.com/article/10.3390/jcm14248668/s1, PRISMA 2020 Checklist.
